# Seroprevalence of Canine Herpesvirus-1 in Breeding Dogs with or Without Vaccination in Northwest Italy

**DOI:** 10.3390/ani10071116

**Published:** 2020-06-29

**Authors:** Ada Rota, Andrea Dogliero, Teresa Biosa, Margherita Messina, Paola Pregel, Loretta Masoero

**Affiliations:** 1 Department of Veterinary Sciences, University of Turin, 10095 Grugliasco, Italy; ada.rota@unito.it (A.R.); paola.pregel@unito.it (P.P.); 2 Department of Natural Reserves & Wildlife Protection, Ministry of Municipality & Environment, Doha 22332, Qatar; 3 Experimental Institute for Zooprophylaxis in Piedmont, Ligura and Aosta Valley, 10154 Turin, Italy; teresa.biosa@izsto.it (T.B.); margherita.messina@izsto.it (M.M.); loretta.masoero@izsto.it (L.M.)

**Keywords:** dog, breeding kennels, canine herpesvirus-1, vaccination

## Abstract

**Simple Summary:**

Canine herpesvirus-1 (CHV-1) infection during pregnancy causes foetal deaths and abortion; puppies may acquire the infection at birth from contact with vaginal and oronasal secretions of the dam and are at high risk of death. When a bitch has antibodies against the virus, her litter is safe. Antibodies are produced either following natural infection or vaccination. In this study, we wanted to assess the prevalence of antibodies in a population of breeding dogs in a region of Northwest Italy and assess the diffusion of herpesvirus vaccination. More than half of the 370 tested dogs (50.3%) were seropositive, i.e., had antibodies against CHV-1. No seropositive dogs were identified in only ten out of 33 kennels. In the vast majority of animals, antibodies resulted from natural infection since only 31 dogs had been vaccinated. More than 40% of the seropositive dogs showed high antibody titres and the number of seropositive dogs was lower in younger animals, not yet been in contact with the virus. Our data show that population immunity exists when CHV-1 is endemic. However, vaccination remains an option because seroprevalence can be highly variable and seronegative pregnant bitches will be at high risk of contracting the infection due to viral circulation.

**Abstract:**

Canine herpesvirus-1 (CHV-1) can cause abortion and foetal and neonatal deaths in the bitch. The reactivation of latent infections with asymptomatic virus shedding represents a mechanism, whereby the virus can persist in a dog population. The aim of this study was to investigate the seroprevalence of CHV-1 in a population of breeding dogs in Piedmont, Northern Italy, and to investigate the distribution of herpesvirus vaccination. The study was carried out in 370 animals that were housed in 33 breeding kennels. Antibodies against CHV-1 in serum samples were measured by means of serum neutralization. Vaccination had been performed in 21.2% of the kennels and 8.4% of the dogs. The overall seroprevalence of CHV-1 was 50.3%. In ten kennels (30.3%), no seropositive dogs were identified. The percentage of seropositive dogs ranged from 7.1% to 100% in positive kennels. More than 40% of the seropositive dogs showed high titres. Sex had no significant effect on either seroprevalence or the category of the serum titre. The number of positive animals was significantly lower in the groups of prepuberal bitches and animals younger than 1.5 years. The majority of younger animals showed very high titres, suggesting recent contact with the virus. Our data show that CHV-1 is a common infection in breeding dogs in Piedmont. Vaccination is rarely performed but might be an option, because, although many animals of breeding age already show high antibody titres, seronegative pregnant bitches will be at high risk of contracting the infection due to viral circulation in kennels where the virus is enzootic.

## 1. Introduction

Canine herpesvirus-1 (CHV-1) is an enveloped double-stranded DNA virus that belongs to the genus *Varicellovirus*, subfamily *Alphaherpesvirinae*, family *Herpesviridae*, which was first isolated from stillborn pups by Carmichael et al. [1]. The virus can cause abortion and foetal and neonatal deaths in the bitch [2] and it has been associated with reproductive problems, such as a low conception rate, embryo resorption, and small litter size [3,4,5]. In adult dogs and puppies older than three weeks, the infection is usually asymptomatic or may cause mild clinical signs in the upper respiratory tract and papulovesicular lesions in the genital mucosae [6]. Direct contact is necessary for transmission, and the virus is quickly inactivated in the environment [2]. Like all herpesviruses, CHV-1 persists in a latent state after infection, located in the trigeminal or lumbosacral ganglia, tonsils, and parotid salivary glands, and in retropharyngeal, hypogastric, and pulmonary lymph nodes [6,7]. The reactivation of latent infections and asymptomatic virus shedding represent sources of infection among susceptible animals and may allow the virus to persist in a dog population [2,6]. CHV-1 is considered to be poorly immunogenic. Neutralizing antibody levels increase after infection and may remain high for two months, while low titres may be detected for at least two years [6]. Serum antibodies are important in pregnant bitches to suppress viremia and prevent transplacental foetal infection; dam immunity is also important for the survival of infected puppies, in which the virus can remain localized in the oropharyngeal region [6]. An inactivated subunit vaccine (Eurican Herpes 205^®^, Merial, Lyon, France) is commercially available and it is specifically indicated for bitches during pregnancy to induce an increase in maternal serum neutralizing antibodies. It has been shown to be effective in protecting pups [8].

The aim of this study was to investigate the seroprevalence of CHV-1 in a population of breeding dogs in the Piedmont region, Northwest Italy, and to assess the diffusion and the effect of herpesvirus vaccination.

## 2. Materials and Methods

### 2.1. Dogs and Samples

The study was carried out in 33 breeding kennels in the Piedmont region, Northwest Italy in 2018. A list of small/medium size breeding kennels (which are the more represented typology in the area) was selected from the directory of the Italian Kennel Club, excluding small and toy breeds. When the breeder agreed to take part into the investigation, the study was carried out in his facility. Attention was paid to have a homogeneous distribution of kennels in the investigated area. The selected kennels housed from three to 15 bitches of reproductive age, which produced from two to 10 litters per year. Thirty-eight different breeds were represented. The minimum age for inclusion in the study was eight months, and 40–60% of the selectable animals were sampled in each kennel. The mean number of dogs tested in each breeding kennel was 7.8 (±6.5) females and 3.4 (±3.3) males, for a total of 370 animals, including 257 females and 113 males. The mean ages (±standard deviations) of the bitches and the male dogs were (4.3 ± 2.9) and (4.8 ± 3.0), respectively, ranging from eight months to 16 years for females and 11 months to 13 years for males. All of the animals were healthy and under veterinary control.

For each animal, the sex, age, oestrous cycle phase, and past vaccination against CHV-1 were recorded. A blood sample (2 mL) was collected by cephalic venipuncture into an 8 mL blood collection tube (Vacuette^®^, Z Serum Sep Clot Activator, Greiner Bio-One North America Inc., NC, USA) and carried to the laboratory at 4 °C within five hours of collection. Serum was separated by centrifugation (3500 rpm/min. for 10 min.) and an aliquot was stored frozen at −20 °C until assayed.

### 2.2. Ethics Approval and Consent to Participate

The study was carried out in accordance with ethical guidelines on animal welfare and all of the procedures were in compliance with the guidelines of the Italian Ministry of Health for the care and use of animals (D.L. 4 March 2014 n. 26 and D.L. 27 January 1992 n.116) and with the European Guidelines on Animal Welfare (Directive 2010/63/EU). Owners’ informed consent was obtained.

### 2.3. Serum Neutralization Test

Sera were inactivated in a water bath at 56 °C for 30 min. Serial two-fold dilutions of the inactivated sera in 25 μL serum-free cell culture medium (MEM Earle, Biowest, Meda, MB, Italy) were performed in a 96-well microtiter plate (Euroclone, Pero, MI, Italy) starting at a 1/2 serum dilution and using duplicate rows of wells for each serum sample to be tested. Serum and cell controls were included in each test. Virus strain 552/79 was used for antibody analysis. A dilution of stock virus containing from 100 to 300 TCID_50_ 25 μL^−1^ was prepared while using serum-free cell culture medium containing antibiotics (100 units/mL penicillin and 0.1 mg/mL streptomycin) and rabbit complement at a final concentration of 10%. A 25 μL aliquot of the diluted stock virus was added to every well containing 25 μL of each serum dilution, except for the test serum toxicity control wells and cell control wells in each plate. A virus back titration of the working dilution of the stock virus was included, while using four wells per ten-fold dilution, in order to confirm the validity of the test results. After gently mixing, the plates were covered and incubated at 37 °C for one hour. A suspension of cells was prepared from 3- to 5-day-old cultures of MDCK cells (MEM Earle +10% foetal bovine serum).

A 50 μL aliquot of the cell suspension (20,000 cells per well) was added to every well, and the plates were incubated at 37 °C under 5% CO_2_. After an incubation period of 48–72 h, the plates were microscopically read to determine the viral cytopathic effect. The validity of the test was confirmed by establishing that the working dilution of the stock virus contained 30–300 TCID_50_ of the virus and that the positive serum controls were within 0.3 log10 units of their predetermined titres. The test serum results are expressed as the reciprocal of the dilution of serum that neutralized the virus in 50% of the wells. Titres equal to or greater than 1:4 were considered to be positive for exposure to CHV1. Antibody titres were categorized in the following classes: 1:4–1:8 weakly positive; 1:64–1:128 positive; ≥ 1:256 strongly positive.

### 2.4. Statistical Analysis

The association of vaccination with both seropositivity and titre category was assessed by Fisher’s exact test or the Chi squared test. The bitches were categorized according to their reproductive condition and cycle phase, as follows: spayed, prepuberal, anestrus, estrus, diestrus, pregnant, or lactating. All of the nonvaccinated animals were categorized according to age in the following groups: <1.5 years; 1.5–3.5 years; >3.5–6 years; >6 years. In the group of nonvaccinated animals, the associations of sex, reproductive condition or cycle phase, and the age category with both seropositivity and the titre category were analysed by Fisher’s exact test or the Chi squared test. A *p* value <0.05 was considered to be significant.

## 3. Results

In ten kennels (30.3%), no seropositive dogs were identified. In the positive kennels (*n =* 23), the percentage of seropositive dogs ranged from 7.1% to 100%. The overall seroprevalence of CHV-1 in the dogs was 50.3%. When considering only positive kennels, the seroprevalence was 62.7%. The use of Eurican Herpes 205^®^ was recorded in seven kennels (21.2%) in a total of 31 dogs (8.4%); more than one year had elapsed since vaccination in 19 dogs, while 12 dogs had been vaccinated more recently. A single dog that had been vaccinated more than one year ago exhibited a positive titre; among the recently vaccinated ones, six dogs were positive (two were strongly positive after six months). Among the five dogs that showed negative results, two were sampled three days after vaccination (Table 1).

**Figure 1:**
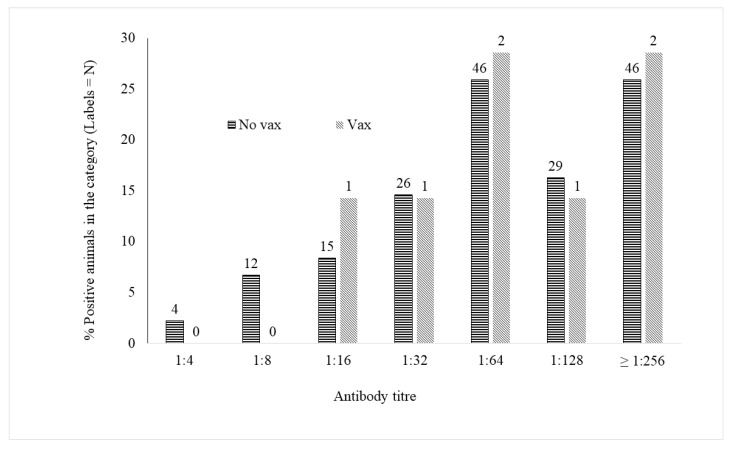
Distribution of CHV-1 antibody titres in the groups of nonvaccinated (No vax; *n =* 178) and vaccinated animals (Vax; *n =* 7). The percentage of animals showing the different antibody titres (1:4, 1:8, 1:16, 1:32, 1:64, 1:128, ≥1:126) appears on the y-axis, while the number in each category is written on the histogram.

Excluding the vaccinated animals, the seroprevalence was 52.5%. Figure 1 shows the distribution of CHV-1 antibody titres in the seropositive nonvaccinated (*n =* 178) and vaccinated (*n =* 7) animals.

More than 40% of the seropositive dogs in both groups showed an antibody titre higher than 1:128, and the percentage rose to approximately 70% when including animals with a titre higher than 1:64. To exclude the effect of vaccination, only data from non-vaccinated animals were analysed. Sex had no significant effect either on seroprevalence or the category of the serum titre.

**Figure 2:**
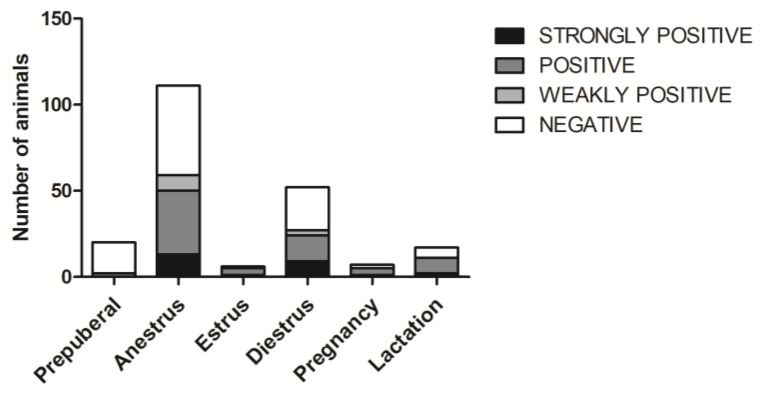
Distribution of serum titres among bitches in different reproductive conditions (Prepuberal, Pregnancy, Lactation) or cycle phases (Anestrus, Estrus, Diestrus). Different grey scales show different titre categories: 1:4–1:8 weakly positive; 1:64–1:128 positive; ≥1:256 strongly positive.

**Figure 3:**
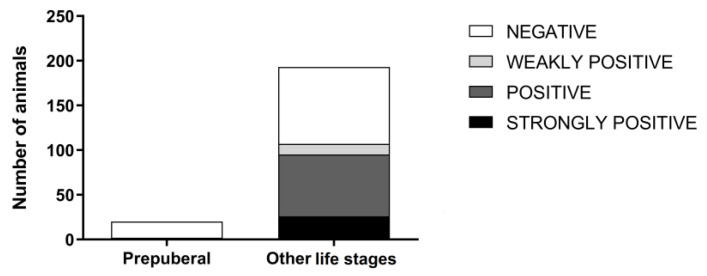
Distribution of serum titres among prepuberal bitches and animals in other life stages. Different grey scales show different titre categories: 1:4–1:8 weakly positive; 1:64–1:128 positive; ≥1:256 strongly positive.

In the bitches, both seropositivity and the titre category were significantly associated with reproductive condition or cycle phase (*p =* 0.0022 and *p =* 0.0381 respectively) (Figure 2). Before puberty, the number of positive animals was significantly lower (*p =* 0.0001), and the titre category was significantly different from those in the post-puberal bitches (*p =* 0.0016) (Figure 3). When analysing both males and females, in accordance with the previous result from prepuberal females, age showed a significant effect on both seropositivity (*p* < 0.0001) and the serum titre (*p* < 0.0001).

**Figure 4:**
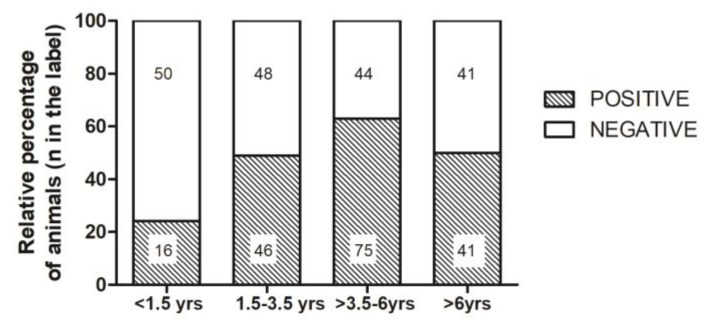
Relative percentage of seropositive (positive) and seronegative (negative) animals in the different age classes (<1.5 years; 1.5–3.5 years; >3.5–6 years; >6 years). The number of animals in each category appears in the histogram.

The group of animals younger than 1.5 years included the highest percentage of seronegative animals (75.8%), and 62.5% of the seropositive animals in this group were strongly positive, which suggested recent contact with the virus (Figure 4).

## 4. Discussion

Group-housed dogs exhibit a higher risk of developing clinical signs of contagious respiratory diseases, and CHV-1 is one of the possible pathogens involved, although it is not a major pathogen of the respiratory tract [9]. Kennel size, hygiene, and kennel cough have been found to be primary contributing risk factors for CHV-1 infection and disease [10]. The impact of CHV-1 on the health and performance of breeding dogs is variable because the virus shows a complex and difficult to predict clinical behaviour [4]. It is a recognized cause of foetal and neonatal deaths, but has also been associated with reproductive disorders, such as embryo resorption, infertility, and low conception rates [4,5]. Latent CHV-1 infection does not show any negative effect on pregnancy, and pregnancy itself does not cause viral reactivation or excretion [11]. Although high antibody titres at the beginning of pregnancy are likely to protect against infertility or embryonic resorption [8], these pathological conditions have also been observed in bitches with high antibody titres [4]. It was suggested that either the pathological events were not related to CHV-1 or that in utero CHV-1 infection following viral reactivation should be considered to be a local phenomenon; in the latter case, cell-mediated immunity in addition to humoural immunity is important to control the evolution of the pathology [4]. When CHV-1 is endemic, immunity exists, although seroprevalence can be highly variable, as observed in our and previous works [3]. Elevated antibody titres can persist for several months, especially in large kennels with more than 20 dogs; recurrent nasal excretion can explain the persistence of high antibody titres [4]. The distribution of the titres that we found in our study suggests that the virus circulates in the kennels and that high titres may be a consequence of frequent reactivation and reinfection. Stress factors can reactivate a latent infection, and oestrus itself is a potential stressor: 85.5% of breeding bitches in proestrus or oestrus were classified as CHV-1 positive in a survey in Norway [12]. CHV-1 is considered to be poorly immunogenic, and antibodies raised against the virus after infection generally decline within a two-month period [6], although low titres can persist for up to eight months or longer [13]. Viral reactivation produces a rapid increase of antibody titres, which peak at 14 days and gradually decrease over a few weeks [14]. In positive kennels, the antibody titres of seropositive dogs were found to remain the same over a 5–18-month period separating two examinations [15].

Our data confirm that serological status is independent of sex, as reported previously [16,17], but is not independent of age. The groups of prepuberal bitches and the males and females younger than 1.5 years showed a significantly lower percentage of seropositive animals. This situation can be explained by progressive seroconversion resulting from repeated contacts among animals during their life. More than 60% of seropositive animals younger than 1.5 years showed a strongly positive titre, which suggested recent contact with the virus. A trend towards an increase in the CHV-1 antibody titre with age has been reported in some studies [3,18], but not in other investigations [16]. Herpesvirus vaccination appeared to be rather sporadic in the breeding kennels included in our investigation. The number of vaccinated dogs was too low to reach univocal conclusions. The fact that the virus was endemic in the kennels means that the effect of vaccination is confounded by the effects of virus circulation and infection/reinfection. High antibody titres six months or one year after vaccination could be the result of recrudescence rather than the persistence of vaccinal antibodies. CHV-1 seems to be enzootic in dogs worldwide. Several studies have investigated the prevalence of CHV-1 in breeding kennels and in dogs in breeding kennels in different countries in past years. The results are highly variable among countries, but the relevant studies are unfortunately old or very old, so the prevalence data are not readily comparable with the present values. The reported percentages of positive kennels ranged from 23% in Switzerland in 1980 [19] to 50% in Belgium in 2001 [20], 26% in Germany in 2004 [15], and 43% in South Africa in 2008 [16]. All of these percentages are lower than our finding of almost 70% positive kennels. Seroprevalence in breeding dogs has been found to be similar to that in owned animals, which has been reported to be approximately 46% in Belgium [21] and to range from 80% in owned dogs to 85.5% in breeding bitches in Norway [12,18]. However, we do not know whether these breeding dogs were housed in kennels, which is a significant condition that is related to virus circulation. A lower but similar seroprevalence between kennel dogs (17.1%) and owned dogs (13.2%) was found in a survey conducted in Southern Italy in 2014, although the number of kennel dogs included was only 16 [22]. In the positive kennels, the mean seroprevalence that we recorded was approximately 63%, which is similar to the percentages that were reported in Belgium in 2001 [20] and in Germany in 2004 [15]. However, the use of different serological methods and varying sensitivities of the applied test can also influence the results. Because our study was focused on small/medium size kennels, the results cannot be straightforwardly generalized to the breeding dog population of the investigated area, and further studies should be necessary to assess the expected effect of different kennel typologies [10].

## 5. Conclusions

Our data show that CHV-1 is a common infection in breeding dogs in Piedmont, although some kennels may be virus-free. In positive kennels, seroprevalence is high and a large proportion of the population shows high antibody titres. In bitches, antibody titres increase after puberty, and it is likely that these animals have protective titres at reproductive age, for pregnancies and newborns. However, vaccination should be taken into account because seronegative pregnant bitches are at high risk of contracting the infection due to viral circulation in kennels where the virus is enzootic. Vaccination could also represent good practice in negative kennels, at risk of virus introduction by latently infected newly entered animals, potentially causing severe reproductive problems in seronegative pregnancies.

## Figures and Tables

**Table 1:** Seroprevalence of CHV-1 in vaccinated (vax) and nonvaccinated (no vax) animals grouped by sex. The percentages of seronegative and positive animals in each category are reported in brackets.

		Negative	Positive	Cytotoxic	Total
Female dog	no vax	109 (48.02%)	118 (51.98%)	0	227
	vax	21 (70%)	7 (23.3%)	2 (6.7%)	30
Male dog	no vax	52 (46.4%)	60 (53.6%)	0	112
	vax	1	0	0	1
